# HPV vaccine in the treatment of usual type vulval and vaginal intraepithelial neoplasia: a systematic review

**DOI:** 10.1186/s12905-018-0707-9

**Published:** 2019-01-07

**Authors:** Stacey Bryan, Cynthia Barbara, Jane Thomas, Adeola Olaitan

**Affiliations:** 1 Research Fellow, Institute for Women’s Health, Gynaecology Cancer Research Centre, 1st Floor Maple House, 149 Tottenham Court Road, London, W1T 7NF UK; 2 University College London Hospitals, 2nd Floor North, 250 Euston Road, London, NW1 2PG UK

**Keywords:** HPV vaccine, VIN, VAIN, Therapeutic vaccine, Vulval disorders, Vulvar intraepithelial neoplasia

## Abstract

**Background:**

HPV DNA is found in almost 80% of VIN/VaIN. Current management is inadequate, with high recurrence rates. Our objective was to review the literature regarding the role of HPV vaccine in secondary prevention and treatment of VIN/VaIN.

**Methods:**

Database searches included Ovid Medline, Embase, Web of Science, The Cochrane Library and Clinicaltrials.gov. Search terms included HPV vaccine AND therapeutic vaccine* AND VIN OR VAIN, published in English with no defined date limit. Searches were carried out with a UCL librarian in March 2018. We included any type of study design using any form of HPV vaccine in the treatment of women with a histologically confirmed diagnosis of VIN/VaIN. We excluded studies of other lower genital tract disease, vulval/vaginal carcinoma and prophylactic use of vaccines. The outcome measures were lesion response to vaccination, symptom improvement, immune response and HPV clearance.

**Results:**

We identified 93 articles, 7 studies met our inclusion criteria; these were uncontrolled case series. There were no RCTs or systematic reviews identified. Reduction in lesion size was reported by all 7 studies, symptom relief by 5, HPV clearance by 6, histological regression by 5, and immune response by 6.

**Conclusions:**

This review finds the evidence relating to the use of HPV vaccine in the treatment of women with VIN/VaIN is of very low quality and insufficient to guide practice. Further longitudinal studies are needed to assess its use in prevention of progression to cancer.

**Electronic supplementary material:**

The online version of this article (10.1186/s12905-018-0707-9) contains supplementary material, which is available to authorized users.

## Background

Vulval/Vaginal Intraepithelial Neoplasia (VIN/VaIN) can precede the development of invasive cancer by a variable period. Usual-type VIN (uVIN) is commonly associated with carcinogenic subtypes of human papillomavirus (most commonly HPV 16) [1]. The HPV attribution for different anogenital lesions varies between studies, however, is found in approximately 76–87% of VIN, 30–40% of vulvar cancer [1], 96% of VaIN and 63–74% of vaginal cancer [2, 3].

The incidence of VIN is thought to be around 1–2/100,000 [4]. Although spontaneous regression has been reported (approx. 1.2%), VIN is considered a premalignant condition (as is VaIN), with a suggested progression rate to cancer of 3–16% [5]. Although primarily seen in older women (over 70 years old), 15% are diagnosed in women under the age of 50. Although presentation of VIN may be asymptomatic, symptoms often include pruritis, burning, and pain which can be distressing [6]. In addition, lesions may be multifocal affecting large areas of the vulva, pigmented or white or present as areas of erythema. Lesions may also be flat, raised or ulcerated [5].

Vaginal Intraepithelial neoplasia is an uncommon (incidence around 0.1/100,000 women in the US) usually asymptomatic disease [7], often associated with other intraepithelial neoplasias. Progression to invasive vaginal cancer has been reported as approximately 2% [8].

The current treatments for VaIN are either surgical (excision or CO_2_ ablation) or medical (Imiquimod, 5FU or vaginal oestrogens) [9]. Most treatment regimens for VIN also consist of ablation or excision, medical therapy with Imiquimod has been shown to be effective [10] and is commonly used by dermatologists and genitourinary medicine (GUM) specialists. Its use can be limited by intolerable side effects such as erythema and vulval pain. Recurrence rates after treatment, regardless of modality are as high as 30–50%, implying that current treatment options are inadequate and not effective in the long term [11]. A review of practice at our unit showed poor response rate to all treatment modalities with rate of progression to invasive disease at 16%. Almost 15% of patients attend for 10 years or more, undergoing multiple biopsies and/or surgical treatments, in addition to being on long term Imiquimod treatment. These women participate in multiple hospital visits which is a burden to them and increases healthcare services costs. These findings from our own practice prompted this systematic review.

Women with natural antibodies to HPV have less frequent recurrence of VIN than those who are antibody negative (22.9% vs 52%) [12]. Three vaccines are available for the primary prevention of HPV infection. A bivalent vaccine (Cervarix® [GSK]) targets high risk HPV types 16 and 18, whilst a quadrivalent vaccine (Gardasil®) targets the same HR HPV types (16 and 18) and two HPV types that commonly cause genital warts (6, 11). A newer recombinant 9-valent vaccine prepared from the purified virus-like particles (VLP) of the major capsid (L1) protein of the 9 HPV subtypes - 6, 11, 16, 18, 31, 33, 45, 52, 58, is now available (Gardasil® 9 [MSD UK]). These are widely used for the prevention of premalignant genital lesions, and premalignant anal lesions, in females and males. These appear to be most effective at preventing CIN in women who are known to be HRHPV negative, but there is moderate to high quality evidence that they reduce high grade CIN for women whose HPV status is not known [13–15]. This may be because prophylactic vaccination has been shown to augment the response to HPV in women who were previously seropositive [16]. This is consistent with studies that indicate that vaccination may have a therapeutic effect in other HPV-associated conditions such as recurrent respiratory papillomatosis (RRP), a benign neoplasm of the larynx among children, caused by HPV 6 and 11 [17].

Prophylactic vaccines act by inducing neutralising antibodies to the viral L1 capsid proteins. It is thought that treatment of already established HPV infection requires activation of T-cells capable of killing virus infected cells, rather than producing antibodies against the virus itself, so therapeutic vaccines (in development), target viral genome E6 and E7 oncoproteins (necessary for initiation and maintenance of transformation to cancer) [18].

Different types of therapeutic vaccines have been tested in other HPV driven diseases. These include;

- Protein-based vaccines, which can be long or short peptide chains, and comprise TA-CIN (Tissue Antigen Cervical Intraepithelial Neoplasia) a fusion protein of HPV 16 L2, E6 and E7 [18, 19];
- DNA based vaccines that induce cytotoxic lymphocytes, T helper cells, and increase B cell immunity;
- A vaccine containing a mixture of two plasmids encoding HPV 16/18 E6 and E7 antigens;
- TA-HPV (therapeutic antigen HPV) a vaccinia vector-based vaccine expressing modified forms of HPV 16 and 18 E6 and E7 proteins and
- Cell-based vaccines such as dendritic cells and tumour infiltrating T-cells [18].

The currently licensed HPV vaccines have an established role in the primary prevention of HPV infection generally in younger populations. The role of the HPV vaccine in secondary prevention and treatment has not yet been fully established. Women with VIN may have symptoms which are recurrent and difficult to treat. Clinicians who treat these patients (dermatologists, GUM specialists and gynaecologists) look for ways to improve the effectiveness of current treatment.

We performed a review of the literature regarding the role of any HPV vaccination for secondary prevention, in the treatment of women with HPV-related VIN and VaIN.

## Methods

We conducted a systematic search of the literature with the strategy to review best available evidence. We searched for systematic reviews, and randomized controlled trials. If these were not available, we would include controlled non-random studies, and uncontrolled studies. We looked into the use of any form of HPV vaccine in the treatment of women with a histologically confirmed diagnosis of HPV related VIN and/or VaIN, versus control/standard treatment. These vaccines included the commercially available licensed vaccines prepared from the VLPs of the L1 protein of common HPV types, or the experimental vaccines against the HPV oncoproteins E6/E7. Our search also included vaccines as adjuvant to usual care. We excluded studies of other lower genital tract disease (pre-invasive or invasive), vulval/vaginal carcinoma and those studies concerning prophylactic use of vaccines.

We excluded studies whose participants were pregnant, immunocompromised, or had a history of allergy to vaccine products. In the included studies, patients with VIN or VaIN were given any form of HPV vaccination.

The outcome measures we wished to evaluate were lesion response to vaccination, symptom improvement, immune response, and HPV clearance. A Core Outcome Set protocol was not used as this is not currently available but is in development for vulva.

Database searches included Ovid Medline, Embase, Web of Science, The Cochrane Library and Clinicaltrials.gov. Search terms included HPV vaccine OR Human Papilloma Virus vaccine OR Papilloma virus vaccines OR HPV Vaccin* AND therapeutic vaccine* AND VIN OR VAIN OR Vulval intraepithelial neoplasia OR Vaginal intraepithelial neoplasia, published in English with no defined date limit. Database searches were carried out with the aid of a UCL librarian in March 2018. Additional papers were identified via the reference lists of included studies (Additional file 1).

## Results

Our search strategy is detailed in the Prisma flow diagram in Additional file 2: Figure S1. Two independent reviewers identified 93 articles after excluding studies with titles unrelated to the subject matter. A further 86 articles were excluded at this stage: 55 were review articles i.e. not primary research or not addressing the review question. Seventeen studies were excluded as they reported on experimental HPV vaccine development - identifying the right dose for effect or analyzing the types of immune response to HPV vaccination. Four studies were concerned with HPV vaccine as prevention not as treatment and three studies were concerned with effect of experimental HPV vaccine in non-human subjects. In a search of clinicaltrials.org we found 4 potential studies – two were active trials but not yet recruiting, with results expected in late 2018 – one involved CIN only and the other, a study from China which had completed, but no results had been reported at the time of writing. Two studies were concerned with other forms of anogenital pre-cancer and 1 study was concerned with trial of Imiquimod only.

There were no RCTs, systematic reviews or controlled studies identified. Seven studies that included 129 women, met our inclusion criteria, the included studies were all uncontrolled case series published between 2000 and 2017 (Table 1).

**Table 1:** Included studies

Author	Study Design	Participants	N	Average Disease Duration	Intervention	Follow up	Results
Baldwin 2003 [20]	Case series	Aged 42–54 (mean age 46) HG HPV positive VIN/VAIN	12	3–14 months	TA-HPV, a live recombinant vaccinia virus, expressing modified versions of the E6 and E7 open reading frames from HPV-16 and HPV-18.	6 months	Enhanced vaccine T-cell specific response (60%) Symptom relief (25%) Lesion response (reduction in size; 83%) Regression to normal histology (8%) HPV negative post intervention (8%) No disease progression over the 6 months follow up period
Daayana 2010 [21]	Case series	Aged 18–70 (mean 46) Biopsy proven VIN2/3	20	7 years	Weeks 1–8 Imiquimod then three intramuscular doses of TA-CIN - E6 and E7 L2 fusion protein - (1 ml of 128 mg/ml) at weeks 10, 14 and 18.	1 year	Lymphoproliferative response (84%) Symptom relief (68.4%) Lesion response (79%) Regression on Histology (63%) HPV negative (74.3%) 1 progression to invasive cancer after the trial period
Davidson 2003 [22]	Case series	HPV16 positive HG VIN	18	6.4 years (6 months – 17 years)	Vaccination with TA-HPV E6/E7 (2.5 × 10^5^ plaque-forming units) by a single dermal scarification to the deltoid region of the upper arm.	6 months	Immune response (94%) Symptom relief (50%) Lesion response (44%) Regression on histology (5.5%) HPV negative (11%)
Fiander 2006 [23]	Case series	Aged 18–65 (median 40) Biopsy proven HG non-cervical ano-genital intraepithelial neoplasia	29	2–120 months	Three prime vaccinations of TA-CIN (533 μg) were given intramuscularly into the deltoid region days 0, 28, and 56 followed by TA-HPV administered by dermal scarification of the deltoid (2.5 × 10^5^ plaque-forming units) on day 72.	6 months	Symptom relief (56%) Lesion response - CR (disappearance of lesion) seen in 1/29 (3%), partial (> 50%) in 5 (17%), stable disease in 18 (62%), progression in 5 patients (17%) Regression on histology (17%) HPV negative (14%) Nil developed invasive lesions Trend for older women to respond Prime boost strategy does not demonstrate additional efficacy compared to the recombinant vaccine (TA-HPV) alone
Kenter 2009 [19]	Case series	Aged 23–61 (mean 29) Histologically confirmed HPV16 positive VIN3	22	3–185 months	Vaccine containing nine HPV-16 E6 and four HPV-16 E7 synthetic peptides, the vaccine was administered subcutaneously at 3-week intervals	1 year	Immune response − 85% had circulating T cells post vaccine Symptom relief (63%) Lesion response (79%) Regression on histology (45%) HPV status – 4/20 (20%) negative at 3 months
Muderspach 2000 [24]	Case series	Median age 29 CIN2/3 or VIN 2/3	16	–	Vaccination HPV-16 E7 12–20 peptide, 3 groups varied dose LEEP as standard in CIN patients	1 month	Immune response 10/16 (63%) Lesion response 9/17 (53%) HPV status 12/18 negative (67%) Symptom relief and histology not reported
Samuels 2017 [25]	Case series	Median age 50 Biopsy proven HPV16 positive uVIN	12	–	TTFC-E7SH, *E. coli* derived plasmid backbone, fusion protein of tetanus toxin, and shuffled version of HPV16 E7 oncoprotein. Patients vaccinated with a fixed dose on days 0,3, and 6 then received booster at week 4. Administration using novel intradermal application (tattooing). Cohort 1–0.2 mg injection, cohort 2 – 2 mg.	3 months	Symptoms – no response after 3 months Lesions – no response after 3 months Histological features – no response after 3 months Vaccine induced T cell responses found in 4/12 patients

### Study participants

Of the 129 participants 126 had VIN and 3 had VAIN [19–25]. Duration of disease (VIN/VAIN) ranged from 2 months to 17 years, with approximately 60% of patients having had previous treatment(s). In two of the studies the women had a mean age of 29 whilst the other studies’ participants were over the age of 40 years. The studies showed heterogeneity in the number of patients included (12 to 29) and disease duration prior to vaccination (2 months to 17 years), whilst the follow up was equally short (1–12 months) in all.

### Intervention

None of the studies used the commercially available vaccines. All of the studies used experimental vaccines of either TA-HPV (therapeutic antigen HPV) a vaccinia-based vector vaccine, or TA-CIN (Tissue Antigen Cervical Intraepithelial Neoplasia), a fusion protein vaccine comprising HPV16 viral proteins L2, E6 and E7 – the latter 2 responsible for inactivation of the tumour suppressor proteins p53 and pRb respectively, leading to hyper-proliferation of host cells and overexpression of p16 and p14 [5], which can lead to cancer.

### Outcomes

Change in lesion size pre and post HPV vaccination was an outcome reported by all 7 studies. Symptom relief was reported by 5 studies, HPV clearance by 6 studies, histological regression by 5 studies, and immune response by 6 of the 7 studies. Follow-up was short - most commonly from 1 month to 1 year.

#### Lesion response to experimental HPV vaccination

Regression of the lesion was assessed by measuring lesion size before and after vaccination, commonly in two dimensions and taking the average of both sizes. Most of the studies categorized these into partial response (if more than a 50% reduction in size), no response (if less than 50% reduction) or complete response if no lesion was visible following vaccination. In these studies, lesion regression varied from no response up to an overall partial and complete response of 83%.

#### Symptom relief

Clinical improvement in symptoms was measured by either direct questioning at each visit or by asking participants to keep a symptom diary. Although no formal validated tool was used, commonly, symptoms were subjectively graded into none/mild/moderate/severe, depending on how it affected patient’s daily lives. The effects on symptom relief varied from no overall change in symptoms to 68.4% of women becoming symptom free after the follow up period.

#### Immune response to experimental HPV vaccination

Immune response to vaccination ranged from 30 to 94%. Various different markers were used to determine immune responses (T-cell responses vs lymphoproliferative vs cytokine). The types of experimental vaccines, schedules and in some cases delivery method, also differed and this may contribute to the heterogeneity seen between study findings.

#### HPV clearance

HPV clearance is defined as testing HPV negative post vaccination having previously tested positive. The rate of HPV clearance varied between 8 and 74%. Histological regression is defined as the index VIN/VaIN lesion being downgraded from a high grade to a low-grade lesion. The rate of this occurring varied from 0 up to 63%. HPV status and histological regression did not seem to correlate with regression of the lesion nor the patient’s symptoms.

There was a wide variation in results in relation to the above outcomes, which may reflect the differences in patients and the types of vaccines and schedules used.

## Discussion

This study is the first systematic review of the use of any HPV vaccination in the treatment of HPV related vulval and vaginal disorders. This review has highlighted that no high-quality studies using commercially available vaccines have addressed this research question - available vaccines are designed for prevention. Studies that have been carried out are small, case series without control groups, rather than randomized controlled trials. They have used experimental therapeutic vaccines rather than the available vaccines. The study designs are of low methodological quality and a high risk of bias. Systematic reviews of randomized control trials indicate that available HPV vaccines are safe and effective for prevention. Effectiveness seems to decrease with age and exposure to HPV. The role of these vaccines as adjuvant to treatment has not been studied. The efficacy and safety of the experimental vaccines is yet to be established and it is likely to be a number of years before therapeutic vaccines become available for clinical use. Overall the evidence to answer the clinical questions of the role of HPV vaccination in the treatment of VIN/VAIN is of very low-quality.

### Clinical responses to experimental HPV vaccine

When comparing other systematic reviews and studies evaluating treatment outcomes for vulval disease, reporting centers primarily on response to treatment, with response being described as resolution, persistence or progression. This could be assessed clinically as a reduction in size of the lesion, symptoms response as reported by the patients, histological resolution (this assessment is invasive because it requires repeat biopsies), HPV clearance or persistence.

A number of patients experienced relief of their symptoms post vaccination. A large number also experienced a reduction in the size of their lesions - it is unclear whether this correlates with symptom relief. Current treatments for VIN may be effective in the short term but with significant side effects and a high risk of recurrence.

Of the studies which we identified, one reported no response in any of the initial outcome measures after 3 months [25]. In this study the novel technique of intradermal tattooing was used. This technique has been evaluated in animal models and the efficacy compared to traditional intramuscular vaccination has not been established in humans. The method of vaccination may have contributed to these negative results.

#### Strengths and limitations

Our review examines the role of HPV vaccination as an adjuvant to treatment for women who have HPV related VIN/VaIN. Whilst Miltz et al have examined the role of vaccination in anogenital pre-cancer [26], our review differs in that it focuses on vulval and vaginal diseases rather than cervical/CIN. We were interested in exploring interventions that could be used alongside existing treatments for VaIN and VIN to improve effectiveness and/or reduce recurrence and need for further treatments. We used a systematic approach to search library databases. None of the studies directly compared a control group with HPV vaccination, all studies found were uncontrolled case series, with short follow up for what is a chronic, long term disease. We were therefore limited by the level of evidence with which to answer the question.

## Conclusion

Women with VIN/VaIN are living with chronic conditions, requiring prolonged and often repeated treatments which can have intolerable side effects, and yet high relapse rates. More effective treatments are needed that will reduce symptoms, reduce progression and reduce the rate of relapse. Having been introduced almost a decade ago as part of the childhood vaccination program, we should begin to see a reduction in the incidence of VIN/VAIN and other anogenital pre-cancers. In the meantime, for patients who have not been vaccinated, newer treatments with fewer side effects and morbidity are warranted. The evidence for the use of HPV vaccination in VIN/VaIN is of low quality and insufficient to guide practice. All of the vaccines used in these non-randomized studies were experimental and we did not find any research evaluating the use of commercially available vaccine as an adjuvant to usual treatment of VAIN/VIN. We aim to deliver such a study following on from this review. More studies are needed to investigate clinically important patient outcomes (symptoms, non-progression and recurrence rates) with the use of HPV vaccine in a therapeutic setting. It would be prudent to also investigate its use in secondary prevention of cancer, which would need to be assessed through further longitudinal studies.

## Additional files

Additional file 1: Appendix S1 – searches. (PDF 516 kb)

Additional file 2: Figure S1 – Prisma Flow Diagram. (DOCX 108 kb)
