# Supplementary material for: HPV vaccine in the treatment of usual type vulval and vaginal intraepithelial neoplasia: a systematic review
Source: BMC Womens Health. 2019 Jan 7;19:3. doi: 10.1186/s12905-018-0707-9 (PMC6323700; doi:10.1186/s12905-018-0707-9)
Supplement: Supplementary file 1 — Appendix S1 – searches. (PDF 516 kb) [file 12905_2018_707_MOESM1_ESM.pdf]

Searches for systematic review

Ovid MEDLINE® Epub ahead of print, In Process & Other Non-indexed Citations, Ovid MEDLINE ® Daily, Ovid MEDLINE and Versions ® 1946 to March 21 2018

Date search commenced Monday 26th March 2018

1. exp Papillomavirus Vaccines/ (6262)
2. HPV vaccin*.mp. [mp=title, abstract, original title, name of substance word, subject heading word, keyword heading word, protocol supplementary concept word, rare disease supplementary concept word, unique identifier, synonyms] (5956)
3. HPV immunisation.mp. [mp=title, abstract, original title, name of substance word, subject heading word, keyword heading word, protocol supplementary concept word, rare disease supplementary concept word, unique identifier, synonyms] (49)
4. HPV inoculation.mp. [mp=title, abstract, original title, name of substance word, subject heading word, keyword heading word, protocol supplementary concept word, rare disease supplementary concept word, unique identifier, synonyms] (1)
5. 1 or 2 or 3 or 4 (8360)
6. VIN.mp. (1046)
7. vulvar intraepithelial neoplasia.mp. (580)
8. vulva* intraepithelial neoplasia.mp. [mp=title, abstract, original title, name of substance word, subject heading word, keyword heading word, protocol supplementary concept word, rare disease supplementary concept word, unique identifier, synonyms] (699)
9. vaginal intraepithelial neoplasia.mp. [mp=title, abstract, original title, name of substance word, subject heading word, keyword heading word, protocol supplementary concept word, rare disease supplementary concept word, unique identifier, synonyms] (259)
10. exp *Papillomavirus Infections/ (23393)
11. vagina* intraepithelial neoplasia.mp. (259)
12. vaginal high grade squamous intraepithelial lesion.mp. (1)
13. vulva* squamous intraepithelial lesion.mp. (4)
14. VAIN.mp. (683)
15. 6 or 7 or 8 or 9 or 10 or 11 or 12 or 13 or 14 (25217)
16. 5 and 15 (4644)
17. therapeutic vaccin*.mp. [mp=title, abstract, heading word, drug trade name, original title, device manufacturer, drug manufacturer, device trade name, keyword, floating subheading word] (3083)
18. 16 and 17 (147)

Studies excluded after reading titles – (78)

    - Animal models
    - Cervix/anal cancer/head and neck
    - Prevention
    - Cancer

- 69 studies identified based on eligibility criteria
    - Review articles (52)
    - Vaccine development (13)
    - Studies excluded after reading abstracts (65)

- **4 studies identified**
    - Full text not available (1)
    - **Daayana 2010 - included**
    - **Van Poelgeest 2016 – excluded as trial of Imiquimod at vaccine site**
    - **Welters 2010 – excluded as study on immunogenicity after 1 vaccine. Follow on from Kenter Study (same group), no clinical measurement**

Embase Classic + Embase. 1980 to 2018 Week 13
Date searched 26th March 2018

1. exp Papillomavirus Vaccines/ (10999)
2. HPV vaccin*.mp. [mp=title, abstract, heading word, drug trade name, original title, device manufacturer, drug manufacturer, device trade name, keyword, floating subheading word] (8034)
3. HPV immunisation.mp. [mp=title, abstract, heading word, drug trade name, original title, device manufacturer, drug manufacturer, device trade name, keyword, floating subheading word] (60)
4. HPV inoculation.mp. [mp=title, abstract, heading word, drug trade name, original title, device manufacturer, drug manufacturer, device trade name, keyword, floating subheading word] (4)
5. 1 or 2 or 3 or 4. (13560)
6. VIN.mp. (1734)
7. vulvar intraepithelial neoplasia.mp. (875)
8. vulva* intraepithelial neoplasia.mp. [mp=title, abstract, heading word, drug trade name, original title, device manufacturer, drug manufacturer, device trade name, keyword, floating subheading word] (1079)
9. vaginal intraepithelial neoplasia.mp. [mp=title, abstract, heading word, drug trade name, original title, device manufacturer, drug manufacturer, device trade name, keyword, floating subheading word] (604)
10. exp *Papillomavirus Infections/ (14809)
11. vagina* intraepithelial neoplasia.mp. (606)
12. vaginal high grade squamous intraepithelial lesion.mp. (2)
13. vulva* squamous intraepithelial lesion.mp. (3)
14. VAIN.mp. (1273)
15. 6 or 7 or 8 or 9 or 10 or 11 or 12 or 13 or 14 (18233)
16. 5 and 15 (2883)
17. therapeutic vaccin*.mp. [mp=title, abstract, heading word, drug trade name, original title, device manufacturer, drug manufacturer, device trade name, keyword, floating subheading word] (4151)
18. 16 and 17 (72)

Studies excluded after reading titles – (63)

- Animal studies
- Review articles
- Vaccine development
- Head and neck/cervix/other cancer

9 studies identified based on eligibility criteria

- Review article (1)
- Studies in mice (1)
- Vaccine development (4)

3 studies identified

- **Daayana 2010 - included**
- **Fiander 2006 – prime boost vaccination**
- **Welters 2010 – excluded see above**

Web of Science 1900 to present, all databases - 26th March 2018

1. (Human papillomavirus vaccines) (13,581)
2. (HPV vaccin*) (13,967)
3. (HPV immunisation) (4,760)
4. (HPV inoculation) (125)
5. #4 OR #3 OR #2 OR #1 (17,592)
6. (VIN) (10,230)
7. (VAIN) (3,507)
8. (vaginal intraepithelial neoplasia) (5,723)
9. (vulval intraepithelial neoplasia) (308)
10. (vulvar intraepithelial neoplasia) (1,941)
11. (papillomavirus infections) (52,395)
12. #11 OR #10 OR #9 OR #8 OR #7 OR #6 (68,830)
13. #12 AND #5 (12,090)
14. (Therapeutic vaccine) (100,933)
15. #14 AND #5 (4,518)
16. #15 AND #13 (3,548)
17. Filtered within search to include VIN/VAIN **(22)**

Studies excluded after reading title – 18

- Review article 6
- Systematic review – no lesions 1
- Not vulva/vagina – 2
- Immunotherapy 1
- Immune response – 2
- Letter -1
- Mice – 2
- Disease review – 1
- Booster vaccination – 2

4 studies identified

- **Samuels 2017 – include**
- **Van Poelgeest 2016 – excluded see above**
- **Daayana 2010 - included**
- **Davidson 2003 - included**

Cochrane library - 26th March 2018

1. HPV Vaccin* and VIN (2)
    a. Review articles VIN
2. HPV vaccin* and VAIN (4)
    a. All prevention (x1 CIN)

Clinical Trials.gov - 26th March 2018

1. VIN/VAIN + HPV vaccination – 4 studies
    a. 2 active not recruiting
    b. 2 completed
        i. 1 has results but is CIN
        ii. Completed August 15 2016, China – no results

Reference list search – 7 studies

- **Baldwin 2003 - included**
- **Coleman 2016 –** excluded CIN and vaccine dose development
- **Davidson 2003 - included**
- **Kenter 2009 - included**
- Manuri 2007 – excluded mouse model
- **Muderspach 2000 - included**
- Zwaveling 2002 – excluded – mouse model

<u>7 studies included in total</u>

- **Daayana 2010 - included**
- **Fiander 2006 – prime boost vaccination**
- **Samuels 2017 – included**
- **Baldwin 2003 - included**
- **Davidson 2003 - included**
- **Kenter 2009 - included**
- **Muderspach 2000 - included**

Duplicates = 5
