# Supplementary material for: HPV vaccine in the treatment of usual type vulval and vaginal intraepithelial neoplasia: a systematic review
Source: BMC Womens Health. 2019 Jan 7;19:3. doi: 10.1186/s12905-018-0707-9 (PMC6323700; doi:10.1186/s12905-018-0707-9)
Supplement: Supplementary file 2 — Figure S1 – Prisma Flow Diagram. (DOCX 108 kb) [file 12905_2018_707_MOESM2_ESM.docx]

Studies identified through database searching
(n = 251)

## Screening

Additional records identified through other sources (bibliographies/reference lists)
(n = 7)

## Identification

Records after duplicates removed
(n = 253)

Studies excluded after reading titles (n=159)

Full text not available (n=1)

Records screened
(n = 253)

## Included

## Eligibility

Full-text articles assessed for eligibility
(n = 93)

)

Review articles (n=55)

Vaccine development (n=17)

Non-human subjects (n=3)

Vaccine as primary prevention (n=4)

Immunogenicity (n=1)

Studies active, not recruiting (n=2)

Complete but no results (n=1)

Not VIN/VAIN (n=2)

Trial of Imiquimod (n=1)

Studies included in review
**(n = 7)**

Number of patients = 129


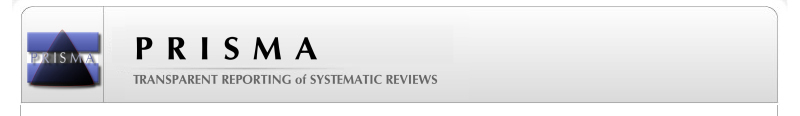


**Systematic Review of Therapeutic HPV Vaccine**
